# Anatomical and functional results after vitrectomy with conventional ILM peeling versus inverted ILM flap technique in large full-thickness macular holes

**DOI:** 10.1186/s40942-023-00509-1

**Published:** 2023-11-14

**Authors:** Adrianna U. Dera, Doerte Stoll, Verena Schoeneberger, Marcus Walckling, Claudia Brockmann, Thomas A. Fuchsluger, Friederike Schaub

**Affiliations:** Department of Ophthalmology, Rostock University Medical Center, Doberaner Str. 140, 18057 Rostock, Germany

**Keywords:** Macular hole, Vitrectomy, ILM flap, ILM peeling, Endotamponade, Surgery, Complication

## Abstract

**Background:**

Aim of the study was to compare success rate and functional outcome following pars plana vitrectomy (PPV) with conventional internal limiting membrane (ILM) peeling versus ILM flap technique for full-thickness idiopathic macular holes (FTMH).

**Methods:**

Retrospective analysis of consecutive eyes with FTMH having undergone vitrectomy with sulfur hexafluoride (SF6) endotamponade 25% at the University Medical Center Rostock, Germany (2009–2020). Eyes were divided according to applied surgical technique (ILM peeling [group P] versus ILM flap [group F]). Inclusion criteria were macular hole base diameters (MH-BD) ≥ 400 μm plus axial length ≤ 26.0 mm. Each group was divided into two subgroups based on macular hole minimum linear diameter (MH-MLD): ≤ 400 μm and > 400 μm. Exclusion criteria were FTMH with MH-BD < 400 μm, trauma, myopia with axial length > 26.0 mm or macular schisis. Demographic, functional, and anatomical data were obtained pre- and postoperatively. Preoperative MH-BD and MH-MLD were measured using optical coherence tomography (OCT; Spectralis®, Heidelberg Engineering GmbH, Heidelberg, Germany). Main outcome parameter were: primary closure rate, best-corrected visual acuity (BCVA), and re-surgery rate.

**Results:**

Overall 117 eyes of 117 patients with FTMH could be included, thereof 52 eyes underwent conventional ILM peeling (group P) and 65 additional ILM flap (group F) technique. Macular hole closure was achieved in 31 eyes (59.6%) in group P and in 59 eyes (90.8%) in group F (p < 0.001). Secondary PPV was required in 21 eyes (40.4%) in group P and in 6 eyes (9.2%) in group F. Postoperative BCVA at first follow-up in eyes with surgical closure showed no significant difference for both groups (MH-MLD ≤ 400 μm: p = 0.740); MH-MLD > 400 μm: p = 0.241).

**Conclusion:**

Anatomical results and surgical closure rate following ILM flap technique seems to be superior to conventional ILM peeling for treatment of FTMH.

## Background

The development of a macular hole is a rare pathology. The incidence varies between 0.02 and 3.3 per 1,000 people over the age of 55 years. Approximately 70% of those are women [1]. Affected patients complain of central visual field loss, distorted vision (metamorphopsia) and reading difficulties. The disease often develops primarily without an identifiable cause. Female sex and increasing age are risk factors [1]. The risk of developing a macular hole increases to 11–15% if there is a macular hole in the fellow eye and there is no posterior vitreous detachment [2–4]. Concerning the etiology and pathogenesis of macular hole, different theories exist. On the one hand, it has been suggested that involutional thinning of the macula is a predisposing factor [5, 6]. On the other hand, vitreoretinal traction plays an important role [7]. In 1988, Gass introduced the first classification of macular holes [8].

The classification into four stages is based on biomicroscopic observations: (1) Early stage is characterized by the appearance of a yellow spot (stage I a) or yellow ring (stage I b) in the fovea. (2) In stage II macular holes, there is a full-thickness foveal defect less than 400 μm in diameter. (3) The fully developed stage III macular hole is a full-thickness defect greater than 400 μm in diameter with posterior vitreous attached. (4) Stage IV macular holes appear similar to stage III holes except that in stage IV holes there is complete posterior vitreous detachment. The new classification published in 2013 by the International Vitreomacular Traction Study (IVTS) group is based on optical coherence tomography results and is divided into vitreomacular adhesion, vitreomacular traction (VMT), and macular hole [9].

Nowadays, due to the development of surgical techniques, macular hole can be effectively treated. The aim of the surgery is to release the traction between epiretinal membrane, retina, and vitreous body. The first surgical treatment of macular hole was described by Kelly and Wendel in 1991 and showed a closure rate of 58% [10]. Eckardt improved this surgical technique by an additional removal of the internal limiting membrane (ILM). Complete closure was achieved in 92% of the patients [11]. However, the closure rate after ILM peeling varies between 70 and 92% in the literature [12, 13].

In 2010, Michalewska described an inverted ILM flap technique for the treatment of large macular holes and high myopia with a 98% closure rate and significantly higher postoperative visual acuity gains. In the inverted ILM flap technique, the ILM is not completely removed from the retina, but is left at the edges of the macular hole. This ILM remnant is then inverted to cover the hole (classic flap) [14]. Alternatively, to minimize iatrogenic trauma associated with ILM -peeling, the ILM can be prepared at the temporal edge of the hole and placed over it (temporal flap). These two surgical techniques have been shown to be equally effective [15].

Very recently, a group of vitreoretinal surgeons and experts in the field of MH surgical treatment (CLOSE Study Group - Classification for Large Macular Hole Studies) proposed an updated surgical classification for large macular holes based on a systematic review of new treatment options including ILM peeling, ILM flaps, macular hydrodissection, human amniotic membrane graft, and autologous retinal transplantation [16]. The authors classified the MH groups according to the preoperative macular hole minimum linear diameter (MH-MLD). The MH size cut-offs were: over 400–535 μm, 536–799 μm, 800–999 μm, and 1,000 μm or larger. ILM peeling showed the best results in MH ≤ 535 μm (closure rate 96.8%), whereas in large MH between 535 and 799 μm ILM flap technique showed better results (closure rate 99.0%). For MH ≥ 800 μm more invasive techniques were required. Furthermore, the authors provided evidence that most MHs over 400 μm in diameter can be closed anatomically with significant visual gains, regardless of their size, chronicity, or previous surgical failures [16].

The aim of this retrospective analysis was to compare two surgical techniques: pars plana vitrectomy (PPV) with SF6 25% endotamponade with conventional ILM peeling versus inverted ILM flap technique in terms of closure rate, visual acuity outcome and number of re-surgeries for idiopathic full-thickness macular holes (FTMH).

## Methods

### Patient data and ocular findings

Retrospective analysis of consecutive eyes with FTMH that underwent vitrectomy at the Department of Ophthalmology, University Medical Center Rostock, Germany, between 2009 and 2020 using electronic patient records.

The study was conducted in accordance with the International Conference on Harmonization for Good Clinical Practice (ICH-GCP) and at all times adhered to the Declaration of Helsinki (2000). Favorable opinion was obtained from the local Institutional Review Board (IRB No. A 2022 − 0124).

**Fig. 1:**
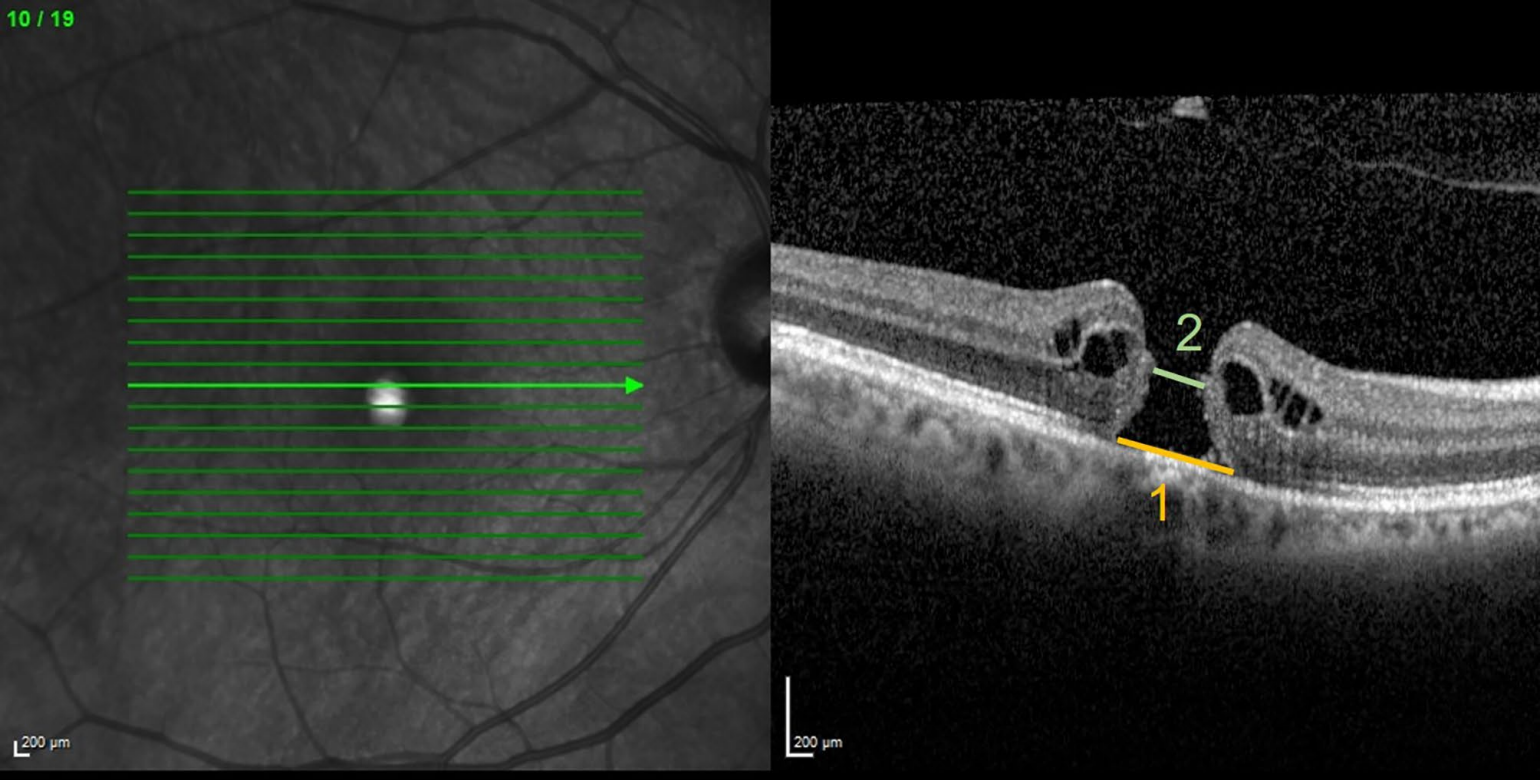
Macular hole characteristics measured by Optical coherence tomography (OCT). Measurement of macular hole size in a representative OCT scan (**1**) at the base of the macular hole (macular hole base diameters (MH-BD)) and (**2**) at the narrowest point (macular hole minimum linear diameter (MH-MLD))

Patients with idiopathic FTMH (stage III and IV according to the classification of Gass et al. [17]) were selected. The diagnosis of the macular hole was confirmed by spectral domain optical coherence tomography (SD-OCT; Spectralis®, Heidelberg Engineering GmbH, Heidelberg, Germany). The specific scan protocol was a custom raster scan pattern with 19 Sect. (512 A-scans each) in a 20°×15° field of view. OCT images were checked for quality and segmentation lines were manually corrected in case of segmentation errors. Two measurements were taken manually in the most central OCT scan showing the FTMH with the largest diameter: the first measurement at the base of the FTMH: macular hole base diameter (MH-BD) and the second at the narrowest point: macular hole minimum linear diameter (MH-MLD) (Fig. 1). Individuals were divided into two groups based on the surgical technique: patients in group P underwent PPV with conventional ILM peeling, while patients in group F underwent PPV with ILM flap technique. SF6 25% was used as endotamponade in both groups. Each group was subdivided according to MH-MLD: ≤ 400 μm and > 400 μm (small and medium versus large MH according to the CLOSE study group updated classification for FTMH [16]).

All participating individuals were operated by two experienced retinal surgeons at the Department of Ophthalmology of the University Medical Center Rostock. Conventional ILM peeling was performed in all patients until 2016. The ILM flap technique was introduced in 2016. Since then, all eyes have undergone this technique.

Patients presented twice for postoperative follow-up: first after at least four weeks, second after approximately 14 months.

At the first follow-up, OCT was used to check for surgical success (anatomical closure of FTMH). If surgical closure was not achieved, re-surgery was scheduled. At each visit, an ophthalmological examination including slit-lamp examination, funduscopy and assessment of best-corrected visual acuity (BCVA) was performed. BCVA was measured in decimal and converted to logarithm of the Minimum Angle of Resolution (logMAR). Lens status was documented at each follow-up examination.

### Inclusion and exclusion criteria

Patients with a large idiopathic FTMH (stage III and IV) with a MH-BD ≥ 400 μm and axial length ≤ 26.0 mm were selected. In pseudophakic eyes, eyes with IOL power < 6.5 dpt were excluded.

Exclusion criteria were foramina with a MH-BD < 400 μm, history of ocular trauma, as well as high myopia with an axial length > 26.0 mm, and macular schisis. Eyes treated with silicon oil tamponade primarily were also excluded.

### Surgical technique

The majority of surgical procedures were performed under retrobulbar anesthesia. General anesthesia was indicated in patients with dementia or anxiety.

The surgical approach was a 23-gauge 3-port PPV (Geuder Megatron S3, Geuder Company GmbH, Heidelberg, Germany) performed by two experienced vitreo-retinal surgeons. Surgical microscope OPMI Lumera 700 with the RESIGHT noncontact visualization system (Carl Zeiss Meditec AG, Jena, Germany) was used. In case of coexisting cataract, PPV was combined with cataract extraction (standard clear cornea bimanual phacoemulsification followed by monofocal posterior chamber, in-the-bag lens implantation). After removal of the vitreous and staining the ILM with Brilliant Peel® (Geuder Company GmbH, Heidelberg, Germany), conventional ILM peeling was performed, followed by complete removal of the ILM with forceps (single-use vitreous forceps endgripping, UNO Colorline, Geuder Company GmbH, Heidelberg, Germany). ILM peeling was performed at the posterior pole up to the major vascular arcade and close to the optic disc. In the ILM flap technique, an ILM flap was prepared from temporal site of the fovea or around the hole as a rosette (classic flap) and left attached to the edges. The macular hole was then covered with ILM. After fluid-air exchange, the position of the flap was checked and corrected if necessary. The vitreous chamber was then flooded with a 25% SF6 air mixture and the trocars were removed. Postoperatively, patients were instructed to keep a head-down position for 3 to 4 days and to avoid the supine position for approximately 3 weeks.

### Statistical analysis

Statistical analysis was performed using IBM SPSS Statistics software (version 28.0.1.0 for Windows, SPSS, Inc, Chicago, IL). For statistical significance testing, tests were selected based on normal distribution of the variables, which was verified using the Shapiro-Wilk test. For all tests, p-values ≤ 0.05 were considered to be statistically significant. Comparability of baseline clinical data between groups P and F was determined using the Mann-Whitney U test and Student’s t-test. Differences in anatomical and functional outcomes were assessed using the chi-squared test. Correlations between the parameters were assessed with analysis of variance ANOVA and Spearman correlation.

## Results

### Baseline parameters and demographic data

Overall 117 eyes of 117 patients with FTMH who underwent PPV at the University Medical Center Rostock between 2009 and 2020 fulfilled the inclusion and exclusion criteria.

There were 14 men and 38 women aged 68.5 ± 8.2 years (range: 42–84 yrs) in group P and 17 men and 48 women aged 69.5 ± 7.7 years (range: 53–89 yrs) in group F. The right eye was operated in 63 patients (group P: 26; group F: 37) and the left eye in 54 individuals (group P: 26; group F: 28).

Axial length was 23.4 ± 0.8 mm in group P and 23.2 ± 1.0 mm in group F. Table 1 shows the demographic data. In both groups, there were no statistically significant differences in terms of age, MH-BD and MH-MLD, preoperative BCVA and axial length.

**Table 1:** Demographic data

Characteristics	Group P *conventional ILM peeling* (n = 52)	Group F *ILM flap* *technique* (n = 65)	p-value
Age ± SD (years), range	68.5 ± 8.2 42–84	69.5 ± 7,7 53–89	*0.892
Male, n (%)	14 (26.9)	17 (26)	-
Right eye, n (%)	26 (50)	37(57)	-
MH-BD, mean ± SD (µm), range	741 ± 207 401–1135	729 ± 212 402–1353	*0.079
MH-MLD, mean ± SD (µm), range	333 ± 114 109–594	373 ± 132 82–740	*0.084
BCVA, mean ± SD (logMAR), range Lens status (phakic, n (%)	0.79 ± 0.25 0.3–1.3 45 (86.5)	0.88 ± 0.31 0.3–1.6 54 (83.1)	**0.180 -
Axial length, mean ± SD (mm)	23.4 (± 0.8)	23.2 (± 1.0)	*0.638

Mean age (at time of surgery in years); range (minimum to maximum); ± standard deviation (SD), MH-BD: macular hole base diameter, MH-MLD: macular hole minimum linear diameter, BCVA: best corrected visual acuity, Shapiro-Wilk test (distribution of variables), *t-test, ** Mann-Whitney U test

In group F, a temporal flap was prepared in 30 cases and a classic flap in 35 eyes. In 4 patients of group F a combined PPV with cataract surgery was performed.

The mean first follow-up time was 44 ± 25 days (group P: 58 ± 31 [range: 27–141], group F: 34 ± 13 [range: 18–91]). The second follow-up was performed in both groups after approximately 14 months (range: 1–80).

### Lens status

In both groups more than 80% of the patients were phakic: 45 (86.5%) in group P and 54 (83.1%) in group F. Pseudophakic status was noted in 7 (13.5%) patients in group P and 11 (16.9%) patients in group F. At first follow-up, lens status changed to pseudophakic in 4 patients in group F who received combined surgery (PPV with phacoemulsification and IOL implantation) and in one patient in group P. At the second postoperative follow-up, 40.3% of eyes in group P and 41.5% in group F were phakic.

### OCT findings – macular hole diameter and closure rate

The preoperative MH-BD of FTMH was 741 ± 207 μm in group P and 729 ± 212 μm in group F (p = 0.079). The MH-MLD was 333 ± 114 μm (range: 109–594 μm) in group P and 373 ± 132 μm (range: 82–740 μm) in group F (p = 0.084).

### Closure rate

Macular hole closure was achieved in 31 eyes (59.6%) in group P and in 59 eyes (90.8%) in group F (p < 0.001) (Table 2). The classic flap was associated with a higher surgical success: out of 6 eyes requiring re-vitrectomy in the flap group, 5 eyes were primarily treated with a temporal flap and one eye with a classic flap. Secondary PPV was required in 21 eyes (40.4%) in group P and in 6 eyes (9.2%) in group F.

**Table 2:** Macular hole closure rate

		Group P *conventional ILM peeling* (n = 52)	Group F *ILM flap technique* (n = 65)	p-value
Macular hole status*	closed, n (%)	31 (59.6%)	59 (90.8%)	**<0.001

*Macular hole status at first follow-up, ** chi-squared test

The macular hole was closed in all patients after the second surgery. In group F, 5 out of 6 eyes were treated again with SF6 endotamponade and one patient with silicone oil endotamponade, whereas in group P, silicone oil endotamponade was used in all patients during re-surgery.

### Functional outcome

Preoperative BCVA was 0.79 logMAR in group P and 0.88 logMAR in group F (p = 0.180).

Postoperative BCVA at first follow-up in eyes with surgical closure and MH-MLD ≤ 400 μm was 0.40 ± 0.25 logMAR in group P and 0.42 ± 0.27 logMAR in group F (p = 0.740); and in eyes with MH-MLD > 400 μm in group P: 0.43 ± 0.04 logMAR and group F: 0.52 ± 0.35 logMAR (p = 0.241).

We observed that the postoperative visual acuity was significantly better in eyes with surgical closure compared to persistent macular holes in both groups (p < 0.001). Visual acuity in patients with surgical closure showed no significant difference between the groups.

**Fig. 2:**
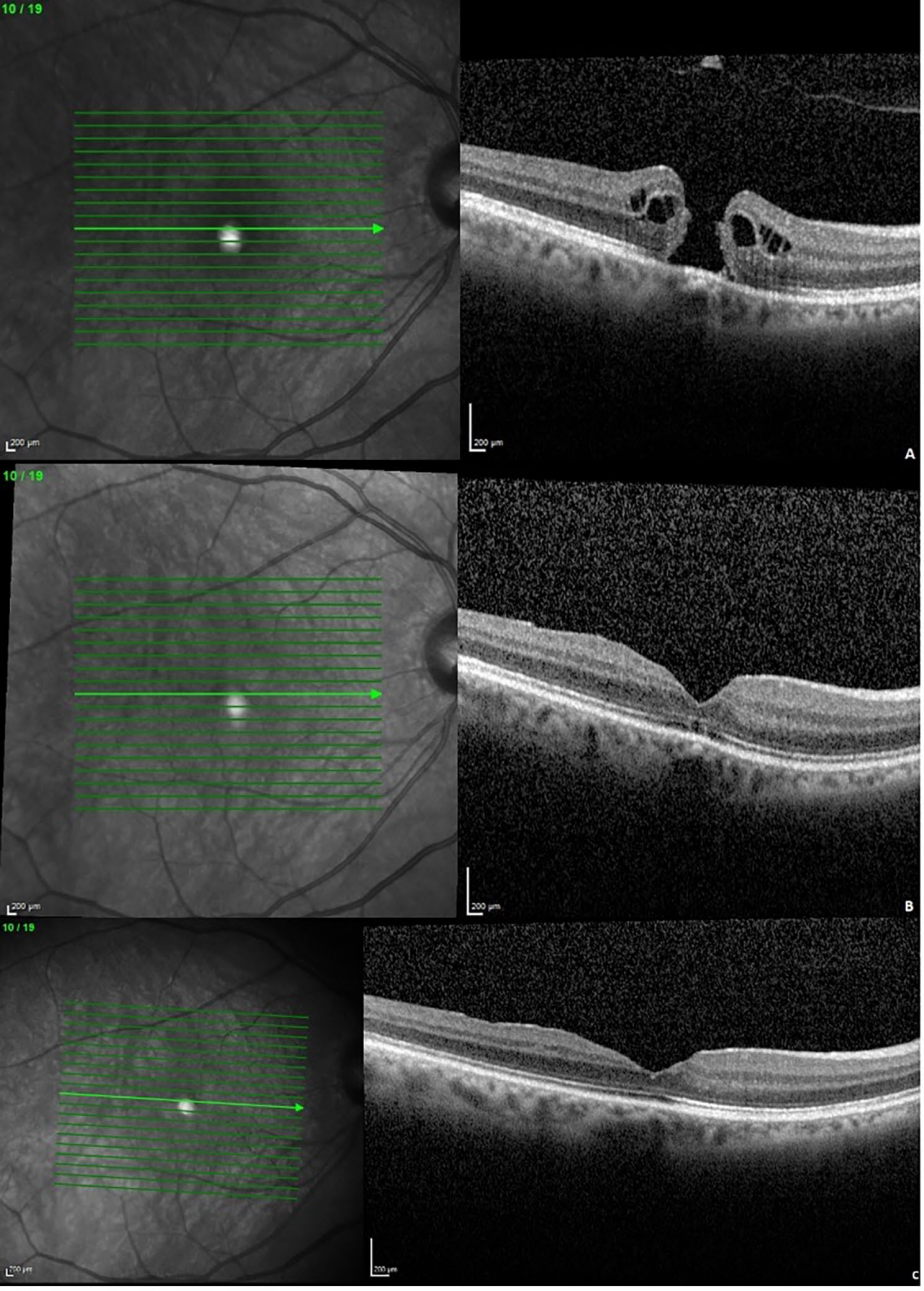
Anatomical results after conventional ILM peeling. Anatomical outcome in an exemplary case following vitrectomy with ILM peeling (**a**) preoperative, (**b**) first follow-up: 6 weeks, (**c**) second follow-up: 3 months

**Fig. 3:**
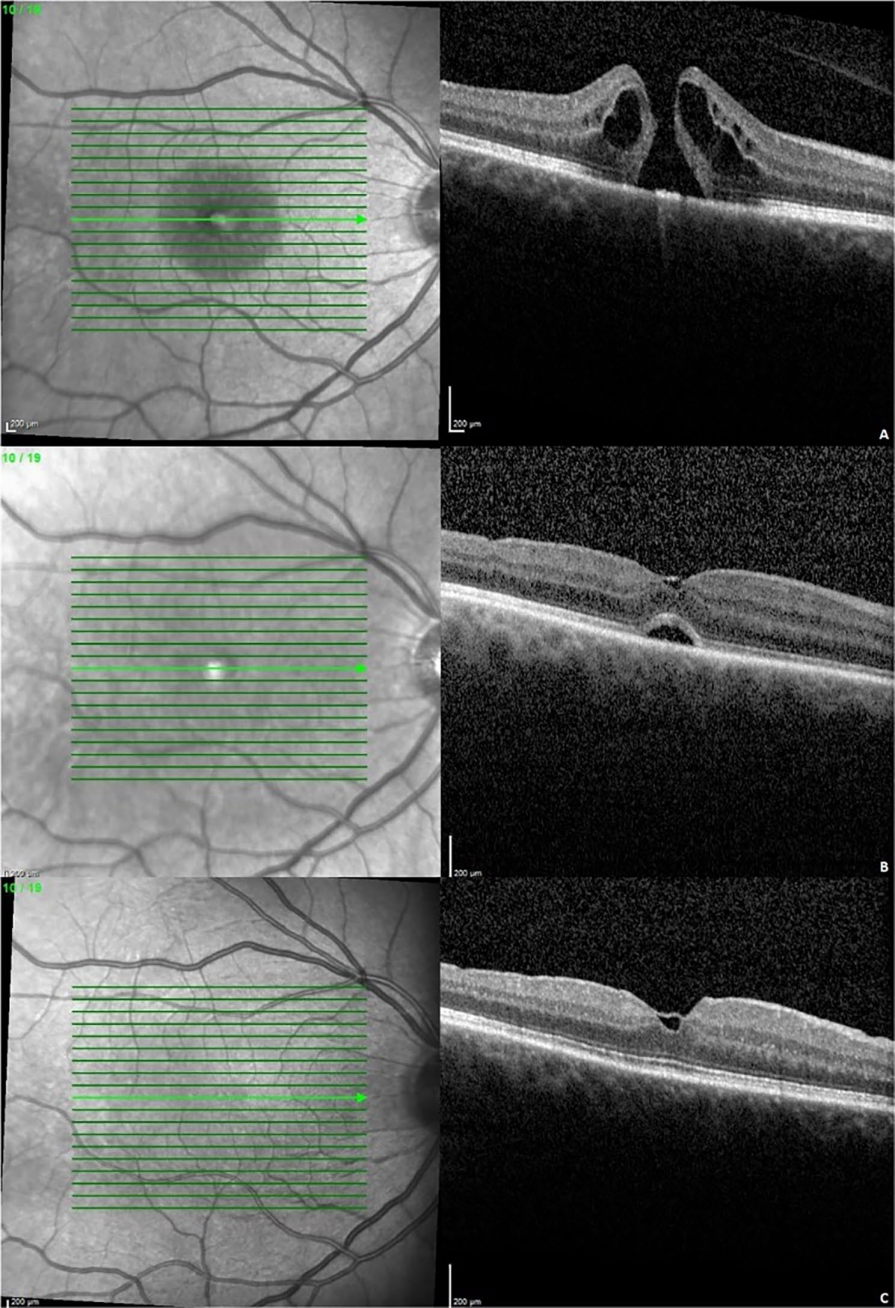
Anatomical results after ILM flap technique. Anatomical outcome in an exemplary case following vitrectomy ILM flap technique (**a**) preoperative, (**b**) first follow-up: 4 weeks, (**c**) second follow-up: 3 months

Visual improvement in eyes with surgical closure was observed not only at first follow-up but also at second follow-up. Figures 2a-c and 3a-c show the OCT findings and morphological changes at first and second visits in one patient after ILM peeling and after ILM flap technique.

### Factors influencing surgical outcome

There was a strong correlation (r = 0.661, p < 0.001; Spearman) between hole size and closure rate in group P and very weak correlation in group F (r = 0.170, p = 0.176; Spearman). In case of conventional ILM peeling, we observed that the larger the hole, the lower the closure rate. The size of macular hole did not affect the anatomical outcome of surgery in the ILM flap group. Table 3 shows ocular findings regarding macular hole size and anatomical outcome. There was a statistically significant association between MH-MLD and pre- and postoperative BCVA (p < 0.001). The larger the hole, the higher the logMAR visual acuity associated with worse pre- and post-operative visual acuity. Axial length ≤ 26.0 mm showed no influence on the closure rate in all included eyes (p = 0.726). Furthermore, gender had no effect on surgical success rate (p = 0.413).

**Table 3:** Ocular findings regarding macular hole size and anatomical outcome

Characteristics	Group P *conventional ILM peeling* (n = 52)		Group F* ILM flap technique* (n = 65)	
MH-MLD (µm)	≤ 400 n = 40	> 400 n = 12	≤ 400 n = 39	> 400 n = 26
Baseline BCVA, mean ± SD (logMAR), range	0.72 ± 0.21 0.3–1.0	1.02 ± 0.23 0.52–1.3	0.8 ± 0.28 0.3–1.3	0.98 ± 0.32 0.4–1.6
Baseline MH-MLD, mean ± SD (µm), range *thereof, closed after first surgery	289 ± 71 109–395 259 ± 63 109–365	598 ± 62 405–594 523 ± 100 452–594	288 ± 80 82–392 285 ± 80 82–392	500 ± 84.4 418–740 490 ± 82 418–740
Macular hole status closed/not closed, n (%)	29/11 (72.5:27.5)	2/12 (16.6:83.4)	35/39 (89.7:10.3)	23/26 (88.4:11.6)
BCVA 1st follow-up, mean ± SD (logMAR), range * thereof, closed after first surgery	0.53 ± 0.32 0–1.0 0.40 ± 0.25 0–1.0	0.96 ± 0.37 0.4–1.56 0.43 ± 0.04 0.4–0.46	0.46 ± 0.29 0.04–1.14 0.42 ± 0.27 0.04–1.14	0.57 ± 0.37 0.06–1.4 0.52 ± 0.35 0.06–1.3
BCVA 2nd follow-up, mean ± SD (logMAR), range * thereof, closed after first surgery	0.52 ± 0.36 0–1.3 0.40 ± 0.27 0–1.0	0.89 ± 0.39 0.22–1.5 0.26 ± 0.05 0.22–0.3	0.40 ± 0.34 0.0–1.3 0.39 ± 0.34 0–1.3	0.53 ± 0.39 0–1.3 0.49 ± 0.36 0–1.3

Range (minimum to maximum), ± standard deviation (SD), BCVA: best corrected visual acuity, MH-MLD: macular hole minimum linear diameter

## Discussion

PPV with inverted ILM flap technique showed a higher closure rate compared to conventional ILM peeling. This surgical procedure is especially a promising method for the treatment of large macular holes such as those included in our analysis. Michalewska et al. first described a 98% closure rate with the flap technique vs. 88% with conventional peeling for large macular holes in a prospective randomized study [14]. The inverted ILM flap technique improved the functional and anatomic outcomes for MH with a diameter greater than 400 μm. In postoperative OCT following inverted ILM flap technique eyes showed improved foveal anatomy compared with the conventional peeling technique [14].In this study, closure of the macular hole was achieved in 90.8% of eyes with the ILM flap technique vs. 59.6% with conventional ILM peeling. Several subsequent comparative studies of these two techniques have demonstrated that the ILM flap technique leads to better anatomical results [12, 18–20]. A large meta-analysis by Shen et al. found a significantly higher closure rate using the ILM flap technique (93% versus 86%) compared to conventional ILM peeling, but no significant difference in visual outcome in large macular holes [21]. The analysis between the classical ILM flap and the temporal flap technique showed that both methods are equally effective [15]. However, Rossi et al. compared the classic ILM flap with the inverted ILM flap and described a slightly better closure rate with a classic flap (100% (n = 13/13) versus 84,6% (n = 12/14)) in large macular holes with a diameter greater than 400 μm [22]. In our evaluation the classic flap was associated with a higher surgical success: out of 6 eyes requiring re-vitrectomy in the flap group, 5 eyes were primarily treated with a temporal flap and one eye with a classic flap.

In our study, the visual outcome in eyes with surgical closure showed no significant difference between conventional ILM peeling vs. ILM-flap technique. In the literature, different results have been reported regarding postoperative BCVA. Some authors described no differences in visual improvement between these two techniques [23]. In other publications, there was a significantly better visual gain after ILM flap surgery [24, 25].

The CLOSE study group could show in a recent meta-analysis of 1,135 eyes with FTMH, that conventional ILM peeling showed superior results in MH ≤ 535 μm with a closure rate of 96.8% and adjusted mean BCVA of 0.49 logMAR. In larger MH with MH-MLD between 535 and 799 μm ILM flap technique showed better results (closure rate 99.0%; adjusted mean BCVA: 0.67 logMAR). For MH with MH-MLD ≥ 800 μm use of human amniotic membrane graft, macular hydrodissection and autologous retinal transplantation showed higher closure rates (100%, 83.3% and 90.5% respectively) [16].

Better visual outcome may be explained by the morphological differences that can be visualized postoperatively in the area of the pre-existing macular hole by OCT depending on the surgical technique. Shiode et al. observed that already 10 days after flap surgery, the macular hole was closed and a proliferation of glial fibrillary acidic protein (GFAP)-positive cells and Müller cells, as well as an increase in neurotrophic factors such as fibroblast growth factor on the ILM surface could be detected in the context of neuronal remodeling [26]. During closure, the ILM serves as a scaffold for cell proliferation and migration, creating a dry environment not surrounded by vitreous fluid. This stimulates the regeneration of retinal cell layer. The postoperative configuration of the macula, especially the outer retinal layers plays a major role in functional success [22].

Increasing axial length is a risk factor for the development of macular hole [27]. A retrospective analysis by Wu et al. showed a significantly higher closure rate in eyes with an axial length ≤ 26 mm compared to eyes > 26 mm [21].

The visual outcome in patients with surgical closure and macular hole diameter (MH-MLD) ≤ 400 μm showed no significant difference between the groups (p = 0.889). In individuals with surgical closure and MH-MLD > 400 μm, there was also no significant difference in postoperative BCVA between these two surgical techniques (p = 0.960).

For eyes with persistent macular hole after first surgery, a closure could be achieved in all cases by second surgery, either using SF6 endotamponade again or silicone oil. Reported closure rates in the literature vary widely regarding this point ranging from 45 − 100% [16, 27–30]. Besides gas tamponade, new techniques including placing free ILM flaps, neurosensory retinal grafts, amniotic membrane, platelet-rich plasma, autologous platelet concentrate, or even lens capsule into larger holes or hydrodissection have been reported with success [16, 28–31].

The limitations of this study include its retrospective design. First follow-up was performed after resorption of the gas tamponade with a mean follow-up time of 44 days. This is relatively late, which is due to the fact that the regular follow-up examinations with the local ophthalmologist were carried out after discharge.

Moreover, the surgical intervention was performed by two surgeons. Besides, new surgical techniques (in this case with ILM flap) depend on learning curve, which may be different for each technique and for each surgeon.

## Conclusions

This study shows that PPV with ILM flap technique is an effective surgical method to treat large macular holes with good anatomical and functional results. The significantly better closure rate compared to conventional ILM peeling suggests a higher efficacy of the treatment. The flap technique should be the preferred surgical procedure in patients with a large full-thickness macular hole.

## Data Availability

The data sets generated during and/or analyzed during the current study are available from the corresponding author upon reasonable request.
